# Sex Differences in Stress and Group Housing Effects on the Number of Newly Proliferated Cells and Neuroblasts in Middle-Aged Dentate Gyrus

**DOI:** 10.3389/fnbeh.2016.00249

**Published:** 2017-01-09

**Authors:** Wen-Yu Tzeng, Hsin-Hua Wu, Ching-Yi Wang, Jin-Chung Chen, Lung Yu, Chianfang G. Cherng

**Affiliations:** ^1^Department of Physiology, National Cheng Kung University College of MedicineTainan, Taiwan; ^2^Institute of Basic Medical Sciences, National Cheng Kung University College of MedicineTainan, Taiwan; ^3^Graduate Institute of Biomedical Sciences, Chang Gung UniversityTaoyuan, Taiwan; ^4^Department of Health Psychology, Chang Jung Christian UniversityTainan, Taiwan

**Keywords:** sex difference, aging, early neurogenesis, dentate gyrus, companions, social buffering, stress

## Abstract

Sex differences in stress and coping responses have been frequently documented in aged people, while whether such differences in aged people may appear at the middle age are unknown. This study was undertaken to study the impact of acute stress and social interaction on early neurogenesis in the dentate gyrus (DG) and hippocampus-related memory in two sexes of middle-aged mice. The number of newly proliferated cells, neuroblasts in DG, the object recognition and location memory in 9-month-old male and female C57BL/6N mice were assessed under baseline conditions as well as following an acute stressor regimen and group housing. Three conspecific companions, serving as “the housing group,” were used to model the social interaction throughout the stressor regimen. Males had lower numbers of newly proliferated cells and neuroblasts under baseline conditions as compared to females. The stressor regimen caused rapid decreases in the number of newly proliferated cells and neuroblasts in female DG but no obvious changes were observed in male DG. Group housing, regardless of companions' age, prevented the stress-induced decreases in the number of newly proliferated cells and neuroblasts in female DG. In contrast, the presence of young or age-matched companions potentiated the stress effect in males by decreasing the number of newly proliferated cells and neuroblasts. Finally, neither the stressor regimen nor group housing affected mouse performances in the object recognition and location memory in either sex. These findings, taken together, provide evidence to support a notion that middle-aged females appear to demonstrate more stress susceptibility on early neurogenesis in DG as compared to middle-aged males, although the hippocampus-related memory performances are comparable and not affected by stress in these males and females. Experiencing stress, middle-aged females are more prone to benefit from social interaction as compared to middle-aged males in this regard. We suggest, accordingly, that involving social interaction may afford a therapeutic advance in preventing stress-produced decreases in early neurogenesis in middle-aged females' DG.

## Introduction

Accrued evidence supports the notion that there are sex differences in the stress susceptibility in aged people (Bangasser and Valentino, 2012). For example, plasma adrenocorticotropic hormone secretion stimulated by a laboratory psychosocial stress protocol is found to be lower in older (mean age over 65 years) as compared to younger (mean age below 24 years) men, an effect not observed in women (Kudielka et al., 2004). Moreover, during a battery of cognitive tasks characterized by challenging aspects of repeated reminders of time remaining and interruptions to correct mistakes, older men (mean age over 65 years) display lower cortisol increases than younger men (mean age below 26 years), while older women exhibit greater cortisol increases than younger women (Seeman et al., 2001). Furthermore, a meta-analysis indicates that psychological stress-mediated cortisol release increases with age more so in women than in men (Otte et al., 2005). Although the findings using experimental animals are less abundant than those in humans, several animal studies demonstrate such age-related sex differences in stress responses and vulnerability (Donahue et al., 2006; Hodes et al., 2009; Kokras et al., 2016). In Rhesus monkeys and rats (mean age over 30 years for Rhesus monkeys and 20 months for rats), the aged brains are found to be significantly more vulnerable to stress-mediated neurotoxicity and the damaging effects of glucocorticoids compared to the younger counterparts' (mean age below 11 years for Rhesus monkey and 3 months for rats) brains in both sexes (Bloss et al., 2013; McEwen and Morrison, 2013). Furthermore, approximately 9.5-week-old female rats have higher basal and repeated restraint stress-stimulated corticosterone levels compared to the age-matched male rats (Hillerer et al., 2013), and such sex differences remain in aged (over 20 months) rats (Bowman et al., 2006).

Stress and stress-induced corticosterone secretion associate negatively with neurogenesis in the dentate gyrus (DG; Gould et al., 1991; Cameron and Gould, 1994; Tanapat et al., 2001; Mattson et al., 2004; Mirescu and Gould, 2006; Pawluski et al., 2009). In an attempt to maximize the stress-induced corticosterone secretion, we have used two stressors, including unavoidable foot shocks and restraint in water, in quick succession (Cherng et al., 2010). Such a tandem stressor regimen may acutely increase serum corticosterone level and rapidly reduce the number of newly proliferated cells and neuroblasts in the DG in adult male and female mouse DG (Cherng et al., 2010; Tzeng et al., 2014). Interestingly, group housing and/or the presence of companions' odors, throughout the stressor regimen may prevent such decreases (Cherng et al., 2010, 2012; Tzeng et al., 2013). A number of studies support the notion that there are sex differences in stress-induced changes in dentate neurogenesis in adult rats and mice (Falconer and Galea, 2003; Westenbroek et al., 2004; Shors et al., 2007). However, whether the adult sex differences in stress-induced changes in dentate cell proliferation and neurogenesis may persist to the middle age and even to the senescence remained unexplored. In addition, it has been documented that the quality of group housing could be related to its stress buffering effects in this regard (Tzeng et al., 2014). Accordingly, the first goal of this study was to assess the likely sex differences on the number of newly proliferated cells and neuroblasts in the DG under baseline conditions as well as following stress alone or housing with a group in 9-month-old (i.e., middle-aged) mice. Although the sex differences have been reported under baseline conditions in the number of newly proliferated cell and neuroblast in adult male Balb/C mice (Tzeng et al., 2014), such sex differences have not been evident in adult C57BL/6 mice (Lagace et al., 2007). Thus, 8-week-old C57BL/6N mice were also used in this study for repeating and extending Lagace et al.'s findings in adult C57BL/6N mice (2007).

A growing body of evidence has shown that local BDNF secretion occurs in parallel with the homeostasis of neurogenesis in the DG and hippocampus-related memory performances (Mustafa et al., 2008; Aimone et al., 2014; Malheiros et al., 2014; Novaes Gomes et al., 2014; Kaptan et al., 2015; Liu et al., 2015; Wang et al., 2016). Since we have previously reported that the stressor regimen used may decrease BDNF secretion in the DG and group housing may prevent such rapid decreases in adult male mice (Tzeng et al., 2013), the second goal of this study was to determine the impact of the stressor regimen and group housing on hippocampus-related, the object recognition and location (Cherng et al., 2007; Kesner et al., 2008; Cai et al., 2012; Wiescholleck and Manahan-Vaughan, 2013; Bustamante et al., 2014), memory performances in the 9-month-old male and female mice.

## Materials and methods

### Animals

Eight-week-old and 9-month-old male and female C57BL/6N mice were obtained from National Cheng Kung University College of Medicine (NCKUCM) Laboratory Animal Center. Mice were group housed by sex in plastic cages (28 × 17 × 12 cm; 4 per cage) in a temperature- and humidity-controlled colony room on a 12-h light/dark cycle with lights on at 07:00 h. It was of importance to note that 8-week-old experimental mice were housed with three age-matched siblings, serving as the group housing companions, for 5 weeks since their weaning. The 9-month-old experimental mice were housed with three age-matched mice, serving as the age-matched companions (or Old Support), for 3 months since their first arrival at 6 months of age. Due to our pilot observations that 6-month-old male mice were prone to display vigorous, sometimes fatal, attacks toward the young male mice when they were housed together, the “Young Support” for the 9-month-old mice were 8-week-old mice from different home cages. Throughout the experiment, mice had access to food (Purina Mouse Chow, Richmond, IN, USA) and tap water *ad libitum*. This study was performed in accordance with the National Institutes of Health Guide for the Care and Use of Laboratory Animals (NIH Publications No. 80-23) revised in 1996. All procedures were approved by the local Animal Care Committee at NCKUCM.

### The stressor regimen

The stressor regimen consisted of two stressors, foot shocks, and restraint in water, in quick succession. Foot shock stressor referred to randomly-scheduled delivery of unavoidable foot shock (0.5 mA and a 1-s duration each with an average of 1 shock/min) for 30 min in a 24-cm long, trough-shaped metal alley (for delivering foot shock). Restraint in water stressor referred to a 30-min restraint in a one third-immersed plexiglas cylinder (7 cm in length and 3.4 cm in diameter for the top circle) within a plastic pan (31 × 23 × 7 cm; Cherng et al., 2010, 2012).

### Object location and recognition tasks

In an attempt to evaluate the correlations between early neurogenesis in the DG and hippocampus-related memory function in 9-month-old mice, mice underwent object location, and recognition tasks at 6–7 h after the conclusion of the stressor regimen or free navigation, approximately the time point that the early neurogenesis in the DG was assayed. Mice received the stressor regimen or free navigation between 09:00 and 11:00. Starting at 16:00, mice underwent object location and recognition tasks. The object location task consisted of a 30-min habituation, 10-min training, and 10-min test trial with the habituation session starting approximately at 6 h after the conclusion of the stressor regimen. Mice first received the 30-min habituation trial free exploring in an empty Plexiglas box (46 cm × 26 cm × 21 cm) with black walls and a bright yellow floor in a dimly lit (<40 lux) test room. Approximately 30 min later, two objects (ceramic tea cups with a bottom diameter of 8 cm placed upside down) were placed in the opposite corners sharing a wall (cup border was 6.8 cm away from the walls) of the box in the same dimly lit test room for a 10-min training trial. In the training trial, mice were allowed to free explore in the box and the time mice spent in exploring two cups was recorded. Since short-term memory was concerned, mice were removed to holding cages for an approximate 10-min retention time following the training trial. As the retention time elapsed, mice were returned to the box with one cup (the one with a lesser time spent in the training trial) moved to a diagonally opposite corner (cup border also 6.8 cm away from two adjacent walls), starting a 10-min test trial. The recognition percentage, ratio of the time spent exploring the tea cup in the new corner over the time spent exploring both tea cups, was used to determine the memory performance in mice from the No Stressor, Stressor, and Stressor and Young Support, Old Support group. It was of importance to note that the box and cups were thoroughly cleaned between adjacent trials to stop the plausible build-up of olfactory cues.

For the object recognition task, mice were first habituated to a dimly illuminated custom-made square Plexiglas box (40 cm × 40 cm × 30 cm) for 30 min. Approximately 30 min later, two equivalent objects (ceramic tea cups with a bottom diameter of 6.3 cm placed upside down) were, then, introduced into the box and being placed on the opposite sides of the box for 10 min. In this 10-min training trial, the time mice spent in exploring these two objects was recorded. Since short-term memory was concerned, mice were removed to holding cages for an approximate 10-min retention time. As the retention time elapsed, mice were returned to the box with one object (the one with a lesser time spent in the training trial) replaced by a novel object (a ceramic tea filter with a top diameter of 6.6 cm placed upside down) for a 10-min test trial. The time that mice spent in exploring the familiar and novel objects was recorded. The recognition percentage, ratio of the time spent exploring the novel object over the time spent exploring both objects, was used to determine the memory performance in mice from the No Stressor, Stressor, Stressor and Young Support, and Old Support group. The box and cups were thoroughly cleaned between trials.

### Grouping and experimental procedures

**Figure 1:**
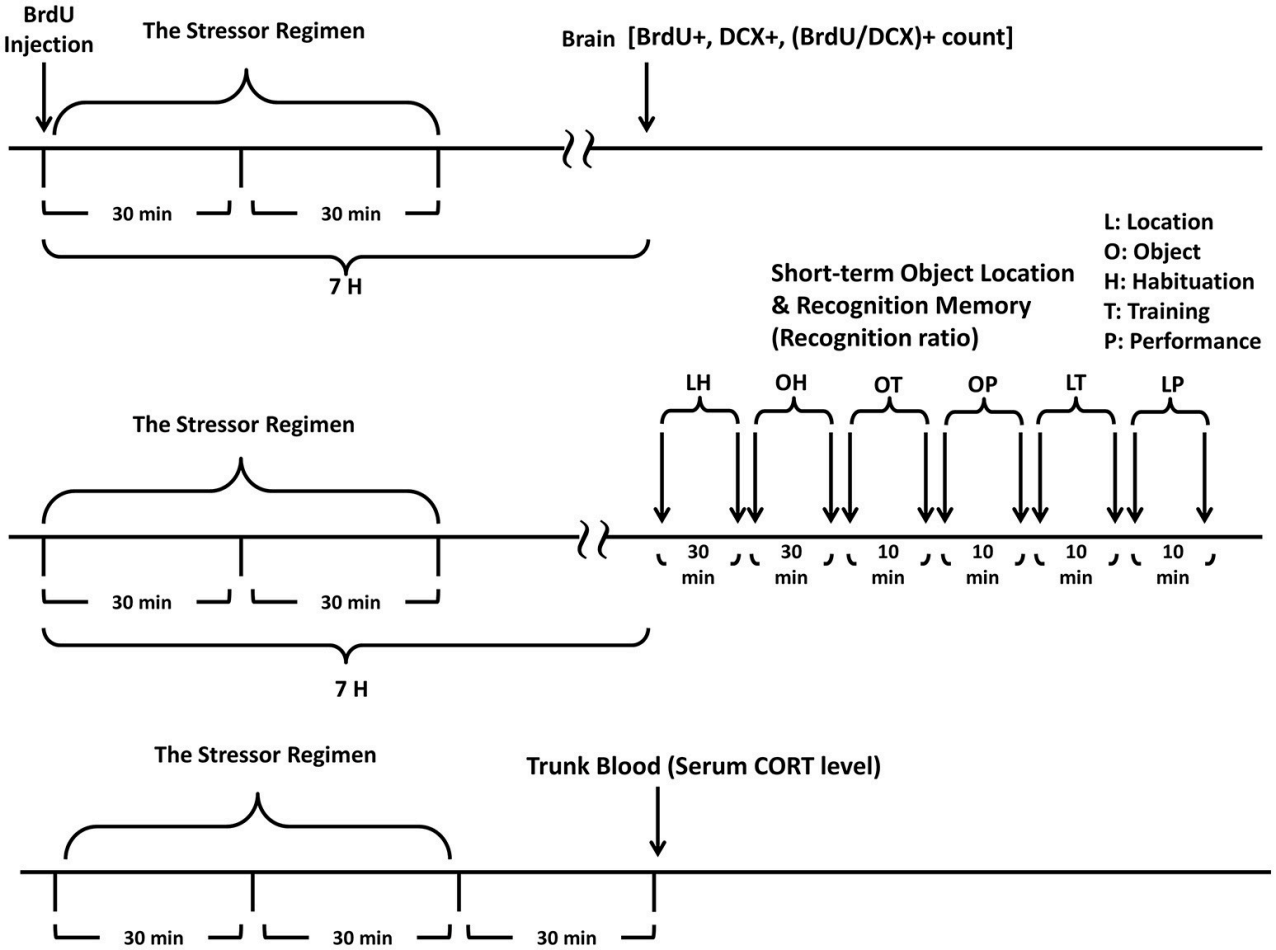
**A schematic representation of experimental procedures**. Timeline for studying the sex differences in the impact of the stressor regimen and group housing on the newly proliferated cell and neuroblast in the dentate gyrus is listed (the top panel). Timeline for studying the impact of the stressor regimen and group housing on the performances of object recognition and location tasks in 9-month-old male and female mice is listed (the middle panel). Timeline for studying the impact of the stressor regimen and group housing on serum CORT levels in 9-month-old male and female mice is listed (the bottom panel). The stressor regimen consists of a randomly-scheduled delivery of unavoidable foot shock (0.5 mA and a 1-s duration each with an average of 1 shock/min) for 30 min in a metal alley immediately followed by a 30-min restraint in a one third-immersed plexiglas cylinder within a plastic pan.

Mice free exploring in the alley for 30 min, plastic pan for 30 min individually and being transferred to a clean plastic holding cage (28 × 17 × 12 cm) alone for 6 h served as control animals (No Stressor group). Other mice received the stressor regimen individually (Stressor group), with 3 same-sex, 8-week-old companions (Stressor and Young Support group), or with 9-month-old (Stressor and Old Support group) companions all followed by a 6-h waiting alone in the plastic holding cage. The timelines for the immunohistochemical staining, object recognition, location memory, and corticosterone assay experiments are listed in Figure 1. The number of mice used in each group for these experiments are summarized in Table 1.

**Table 1:** **A summary of the number of mice used for each group in four experiments**.

**Experiment**	**Sex**	**No stresser**	**Stresser**	**Stresser and young support**	**Stresser and old support**
Early Neurogenesis in 8-week-old	Male	5	5	5	
	Female	5	5	5	
Early neurogenesis in 9-month-old	Male	8	8	7	7
	Female	8	8	7	7
Object Recognition, Location Memory in 9-month-old	Male	10	10	10	10
	Female	10	10	10	10
Corticosterone Assay in 9-month-old	Male	6	6	6	6
	Female	6	6	6	6

*The number is the number of mice used*.

Three groups of 8-week-old C57BL/6N mice (*N* = 15 for males and *N* = 15 for females) for each sex were used to examine plausible sex differences in the impact of stress and group housing on new cell proliferation and early neurogenesis in young adult DG. Five male and female mice served as controls (No Stressor group). Five mice from each sex underwent the stressor regimen individually (Stressor group) and the remaining five mice from each sex underwent the stressor regimen with same-sex, age-matched companions (their cage mates; Stressor and Young Support; Table 1). In an attempt to evaluate sex differences in basal, stress- and group housing-modulated number of newly proliferated cells and neuroblasts in middle-aged DG, 9-month-old male (*N* = 30) and female (*N* = 30) mice were used. Each sex of mice was subdivided into four groups. One group of mice (*N* = 8 for each sex) underwent free navigation (No Stressor), while the other three groups (*N* = 22 for each sex) received the stressor alone (*N* = 8 for each sex, Stressor), with 8-week-old (*N* = 7 for each sex, Stressor and Young Support), or with 9-month-old (*N* = 7 for each sex, Stressor and Old Support) companions (Table 1).

In order to examine the impact of stress and group housing on the hippocampus-related memory performances, 9-month-old female (*N* = 40) and male (*N* = 40) mice were used. Each sex of mice was subdivided into four groups. One group (No Stressor) underwent a free navigation in the alley and the plastic pan 6 h prior to the object location and recognition tasks (*N* = 10 for each sex). The other three groups experienced the stressor regimen individually (Stressor group, *N* = 10 for each sex), with 8-week-old companions (Stressor and Young Support group, *N* = 10 for each sex), or with 9-month-old companions (Stressor and Old Support group, *N* = 10 for each sex; Table 1).

To parcel out the possibility that group housing may prevent the stress-induced decreases in the number of newly proliferated cells and neuroblasts in female, but not male, DG by distinctively altering the stressor regimen-stimulated corticosterone (CORT) secretion in female and male mice, four groups (six mice for each group) of 9-month-old, male and female mice were used. Six mice from each sex free exploring in the alley and plastic pan as aforementioned served as controls (No Stressor group). The remaining 18 mice from each sex received the stressor regimen in the alley and plastic pan individually (Stressor group, *N* = 6), with three 8-week-old, same-sex companions (Stressor and Young Support group, *N* = 6) or with 9-month-old, same-sex (Stressor and Old Support group, *N* = 6) companions (Table 1). Mice were killed at 30 min after the conclusion of the stressor regimen and their trunk blood samples were obtained for serum CORT assay.

### Immunohistochemical staining protocol and quantification of newly proliferated cells and neuroblasts in the DG

Bromodeoxyuridine (BrdU) staining was used to indicate the number of newly proliferated cells and BrdU co-staining with doublecortin (DCX), a microtubule-associated protein expressing exclusively in neuroblasts, was used to reveal the number of early differentiated neuroblasts. It was of importance to note that a BrdU and DCX co-labeling spot was regarded as a newly proliferated neuroblast by using the standard that there was a lack of dendritic process or the presence of short dendritic process with the longest length <five-fold wide of the BrdU-staining spot. An intraperitoneal injection of freshly prepared BrdU solution at a dose of 100 mg/kg (Sigma Chemical, St. Louis, MO, USA; 10 mg BrdU dissolved in 1 ml saline) was given to mice immediately before the beginning of the 1-h stressor regimen. Mice received the BrdU injection between 09:00 and 11:00. Around 16:00, 6 h after the conclusion of the stressor regimen or free navigation in the pan, mice were deeply anesthetized with sodium pentobarbital and transcardially perfused with ice-cold 0.1 M phosphate-buffered saline (PBS, pH adjusted to 7.4), followed by 4% paraformaldehyde in ice-cold 0.1 M PBS. Their brains were removed and postfixed in a 4% paraformaldehyde solution overnight at 4°C and subsequently cryoprotected in 30% sucrose solution for 48 h at 4°C. Coronal sections at 20 μm in thickness were made using a microtome (Shandon Cryotome E, Runcorn, Cheshire, UK). Brain slices were incubated in 50% formamide/2x SSC (sodium chloride/sodium citrate) buffer for 2 h at 65°C, rinsed with 2x SSC, and incubated in 2N hydrochloric acid for 30 min at 37°C. Slices were then rinsed with PBST (triton-100-containing PBS, 1%) buffer, and incubated in a blocking buffer (BSA:240 g, goat serum:160 ≤ gl, sheep serum: 160 ≤ gl in 8 ml PBS) for 2 h at room temperature. The slices, then, were stained by mouse antiBrdU (1:200, Chemicon, Temecula, CA, USA) for newly proliferated cells. For co-staining purpose, brain slices were immunostained with respective primary antibodies [rabbit anti-doublecortin (DCX), 1:200, Cell Signaling Tech, Beverly, MA, USA] for immature neurons and incubated either with Rhodamine (TRITC) conjugated goat anti-rabbit (1:200, Chemicon, Temecula, CA, USA) or FITC conjugated sheep anti-mouse (1:200, Millipore, North Ryde, Australia) secondary antibodies and imaged with a Zeiss fluorescent microscope. Since staining was quantified in the granular and subgranular zone (hilus not included) of the dorsal region of the DG (bregma: approximately −1.30 to −2.30 mm), an average of 50 coronal sections was obtained for each mouse. Using a stereological method, the total number of BrdU- and BrdU/DCX-positive cells in a series of every 7th section spaced at 120 ≤ gM was obtained and then divided by the slice selection ratio (i.e., 7/50) to obtain the estimated total number of labeled cells for the defined range of the dentate gyrus. Cells were counted by a rater blind to the grouping.

### Serum corticosterone assay

In an attempt to determine group differences in serum corticosterone (CORT) level, 9-month-old male mice were killed individually and their trunk bloods were collected 30 min after the cessation of the stressor regimen in another room adjacent to the one they received the stressor regimen. Trunk blood samples were collected in vials and placed at room temperature for 20 min. Blood samples were, then, centrifuged at 4°C for 10 min (1000 g) and serums were obtained, immediately frozen (−80°C) until assay. Serum CORT concentrations were determined by using a CORT enzyme-linked immune-sorbent assay kit (Cayman Chemical Co, Ann Arbor, MI, USA) according to the manufacturer's protocols and an ELISA reader (MULTISKAN EX, Thermo Electron Corp., Finland). The intra-assay variability was 6.6%.

### Statistical analysis

Two-way (sex × treatment) ANOVAs were employed to analyze the sex (male vs. female) and treatment-induced [No Stressor vs. Stressor vs. Stressor and (Young or Old) Support] differences in the number of newly proliferated cells and neuroblasts in the DG, the object recognition and location memory performance in 8-week and 9-month-old mice followed by Bonferroni's *post-hoc* tests if appropriate. Likewise, a two-way ANOVA was used to analyze the sex and treatment differences in serum CORT levels in the 9-month-old mice. And Bonferroni's *post-hoc* tests were used to further reveal specified inter-group differences. The levels of statistical significance were set at *p* < 0.05.

## Results

### No sex differences or obvious effects of stress, group housing on the number of newly proliferated cells, neuroblasts in the DG of the 8-week-old mice

**Figure 2:**
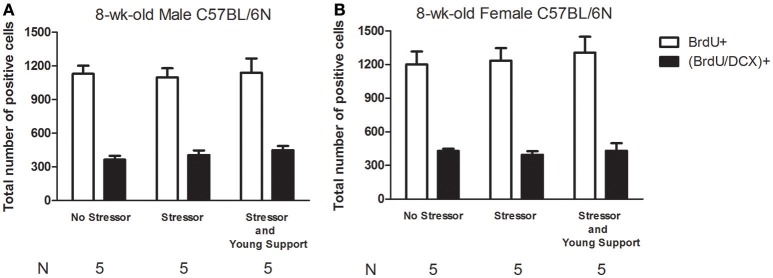
**Sex differences in basal, the stressor- and group housing-modulated number of newly proliferated cell and neuroblast in the dentate gyrus of 8-week-old C57BL/6N mice. (A)** Stressors and the presence of same-sex, age-matched companions did not affect the basal number of newly proliferated cell or neuroblast in the dentate gyrus in 8-week-old male mice. **(B)** Stressor and the presence of same-sex, age-matched companions did not affect the basal number of newly proliferated cell or neuroblast in the dentate gyrus in 8-week-old female mice. No Stressor group represents the mice undergoing free navigation in the alley and pan, while Stressor group stands for the mice receiving the stressor regimen. Young Support is the presence of the same-sex, age-matched mice. BrdU is a short for bromodeoxyuridine, while DCX is a short for doublecortin.

Three groups of 8-week-old mice were used for each sex (Table 1). The “No Stressor” group underwent free exploration in the alley and pan, while the “Stressor” and “Stressor and Young Support” group received the stressor regimen individually and with an age-matched housing group, respectively. Six hours after the conclusion of the stressor regimen or free exploration, mice were killed and their brains were dissected for immunohistochemical staining experiment (Figure 1). Two-way ANOVAs revealed that no sex differences were observed in the baselines in the number of newly proliferated cells [*F*_(1, 24)_ = 1.932, *p* = 0.1773] or neuroblasts [*F*_(1, 24)_ = 0.1336, *p* = 0.7179] in the DG in 8-week-old C57BL/6N mice (Figures 2A,B), indicating that adult C57BL/6N mice do not exhibit sex differences in new cell proliferation or early neurogenesis in their hippocampal dentate gyri. Likewise, the stressor regimen and group housing did not modulate the baselines in the number of newly proliferated cells [*F*_(2, 24)_ = 0.1735, *p* = 0.8417] or neuroblasts [*F*_(2, 24)_ = 0.688, *p* = 0.5122] in the DG in these mice (Figures 2A,B, **4**).

### Sex differences and the impact of stress and group housing on the number of newly proliferated cells, neuroblasts in the DG in the 9-month-old mice

**Figure 3:**
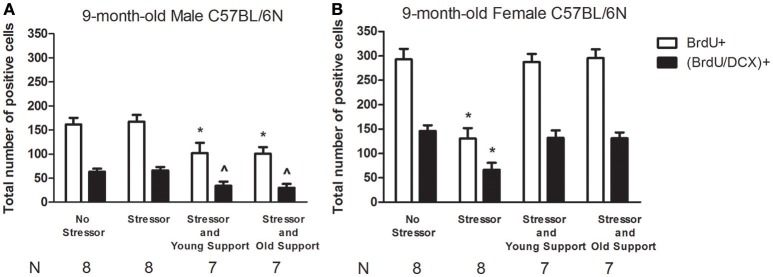
**Sex differences in basal, the stressor- and group housing-modulated number of newly proliferated cell and neuroblast in the dentate gyrus of 9-month-old C57BL/6N mice. (A)** The stressor regimen did not affect the basal number of newly proliferated cell or neuroblast in the dentate gyrus in 9-month-old male mice, while the presence of the same-sex, young or old companions caused significant decreases in the number of newly proliferated neuroblast in the dentate gyrus in 9-month-old male mice. ^*^Significantly lower than the Stressor group. ^∧^Significantly lower than the No Stressor and Stressor group. **(B)** The stressor regimen caused significant decreases in the number of newly proliferated cell and neuroblast in the dentate gyrus in 9-month-old female mice. In contrast, the presence of same-sex, young, or old companions prevented such decreases in these 9-month-old female mice. ^*^Significantly lower than the other three groups. No Stressor group represents the mice undergoing free navigation in the alley and pan, while Stressor group stands for mice receiving the stressor regimen. Young Support and Old Support refer to the presence of three same-sex, 8-week-old, and 9-month-old companions, respectively. BrdU is a short for bromodeoxyuridine, while DCX is a short for doublecortin.

**Figure 4:**
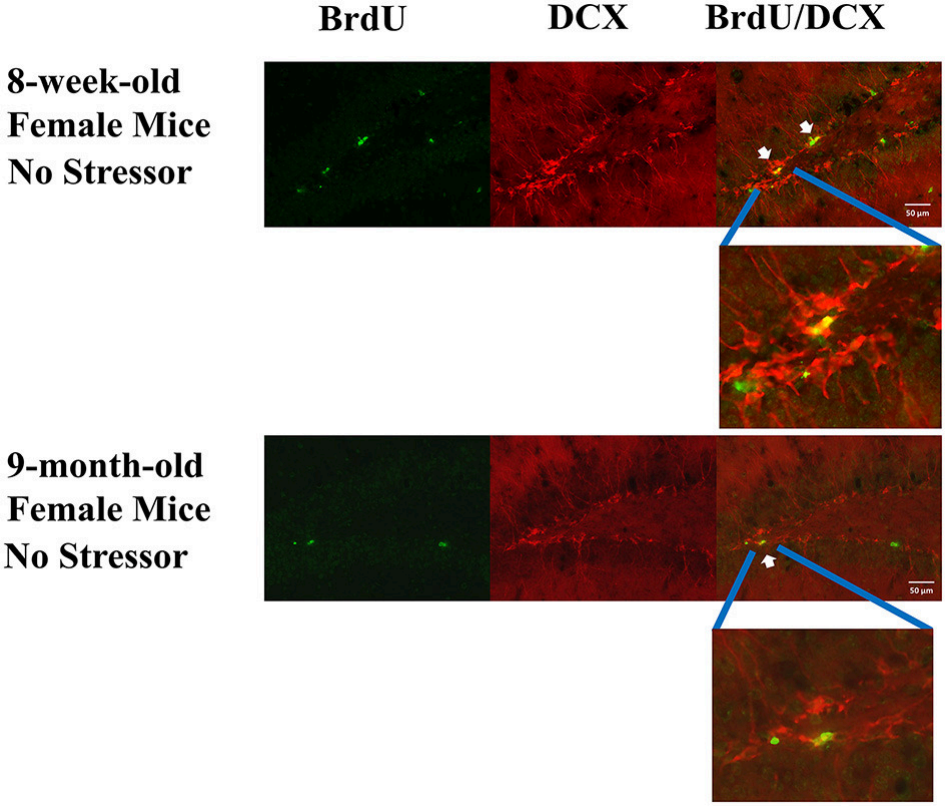
**Representative photomicrographs of the BrdU (green), DCX (red), and BrdU/DCX (yellow)-labeled cells in 8-week-old and 9-month-old female mouse DG without exposure to the stressor regimen**. BrdU is a short for bromodeoxyuridine, while DCX is a short for doublecortin. White arrows point to some of the BrdU and DCX co-labeled cells.

Four groups of 9-month-old mice were used for each sex (Table 1). The “No Stressor” group underwent free exploration in the alley and pan, while the “Stressor,” “Stressor and Young Support,” and “Stressor and Old Support” group received the stressor regimen individually, with an 8-week-old, and 9-month-old group, respectively. Six hours after the conclusion of the stressor regimen or free exploration, mice were killed, and their brains were dissected for immunohistochemical staining experiment (Figure 1). A two-way ANOVA showed that the main effect of sex on the number of newly proliferated cell in the DG was noticed [*F*_(1, 52)_ = 87.12, *p* < 0.0001]. Moreover, there was an interactive effect of sex and treatment on the number of newly proliferated cell in the DG [*F*_(3, 52)_ = 18.39, *p* < 0.0001]. *Post-hoc* tests further revealed that 9-month-old male mice had a lower baseline in the number of newly proliferated cell in the DG as compared to the 9-month-old female mice. Likewise, a two-way ANOVA indicated that the main effects of sex [*F*_(1, 52)_ = 84.02, *p* < 0.0001] and treatment [*F*_(3, 52)_ = 4.63, *p* = 0.0061] on the number of newly proliferated neuroblasts in the DG were evident. Moreover, there was an interactive effect of sex and treatment on the number of newly proliferated neuroblasts in the DG [*F*_(3, 52)_ = 9.891, *p* < 0.0001]. *Post-hoc* tests further revealed that 9-month-old male mice had a lower baseline in the number of newly proliferated neuroblasts in the DG as compared to the age-matched female mice. These analyses, taken together, suggest that there are sex differences in the baseline in the number of newly proliferated cells and neuroblasts in the DG of the 9-month-old C57BL/6N mice (Figures 3A,B). For 9-month-old male mice, the stressor regimen did not affect the number of newly proliferated cells or neuroblasts in the DG, while the presence of young and old companions potentiated the stress effect by decreasing the number of newly proliferated cells and neuroblasts (Figure 3A). In contrast, the stressor regimen significantly decreased the number of newly proliferated cells and neuroblasts in the DG of the 9-month-old female mice (Figures 3B, 4). Interestingly, the presence of young and old companions throughout the stressor regimen prevented such stress effects in these 9-month-old female mice (Figure 3B).

### No sex differences or impact of stress, group housing on the object location, recognition memory performances in the 9-month-old mice

**Figure 5:**
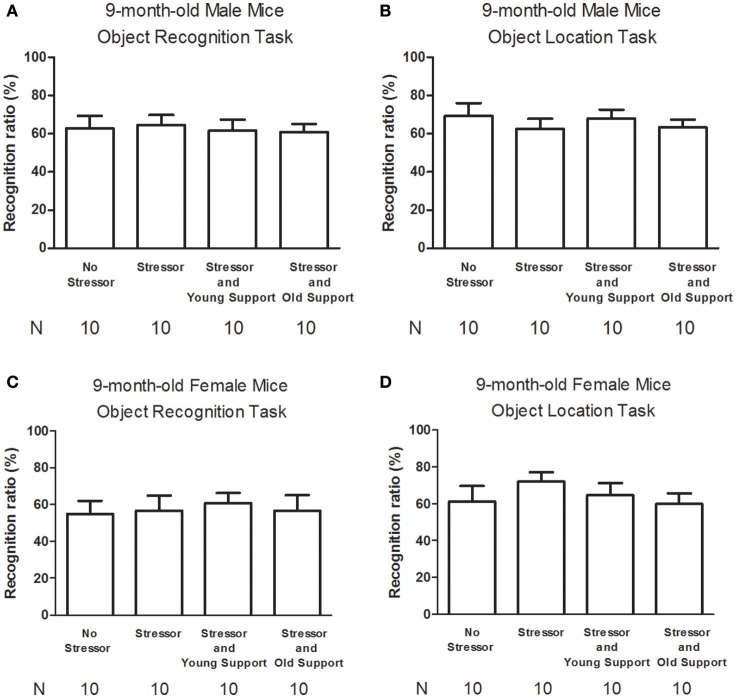
**The modulating effects of the stressor regimen and the presence of companions on the object recognition and location memory performances in 9-month-old male and female mice. (A)** The stressor regimen or the presence of companions did not affect the object recognition memory performances in male mice. **(B)** The stressor regimen or the presence of companions did not affect the object location memory performances in male mice. **(C)** The stressor regimen or the presence of companions did not affect the object recognition memory performances in female mice. **(D)** The stressor regimen or the presence of companions did not affect the object location memory performances in female mice. No Stressor group represents the mice undergoing free navigation in the alley and pan, while Stressor group stands for mice receiving the stressor regimen. Young Support and Old Support refer to the presence of three same-sex, 8-week-old, and 9-month-old companions, respectively.

Four groups of 9-month-old mice were used for each sex (Table 1). The “No Stressor” group underwent free exploration in the alley and pan, while the “Stressor,” “Stressor and Young Support,” and “Stressor and Old Support” group received the stressor regimen individually, with an 8-week-old, and 9-month-old group, respectively. Approximately 6–7 h after the conclusion of the stressor regimen or free exploration, mice received object location and recognition habituation, training, and test (Figure 1). Using two-way (sex × treatment) ANOVAs, no main effects of sex [*F*_(1, 72)_ = 0.0913, *p* = 0.7634; *F*_(1, 72)_ = 1.199, *p* = 0.2772], treatment [*F*_(3, 72)_ = 0.3302, *p* = 0.8035; *F*_(3, 72)_ = 0.0698, *p* = 0.9759], or sex-treatment interactive effects [*F*_(3, 72)_ = 0.7814, *p* = 0.5082; *F*_(3, 72)_ = 0.1298, *p* = 0.9421] on the object location or recognition performances were observed in the 9-month-old mice. The stressor regimen or the presence of young or old companions did not seem to affect the object recognition or location performances at ~7 h after the conclusion of the stressor regimen in male (Figures 5A,B) or female (Figures 5C,D) mice.

### Serum CORT levels at 30 min following the conclusion of the stressor regimen in 9-month-old mice

**Figure 6:**
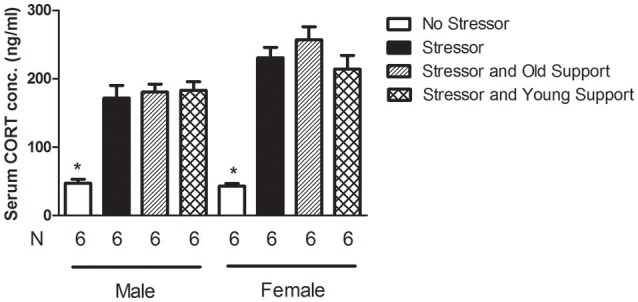
**The serum CORT levels following the stressor regimen in 9-month-old male and female mice**. The stressor regimen enhanced serum CORT levels, while the presence of either young or old support did not affect such stressor regimen-enhanced CORT concentrations. ^*^Significantly lower than the other three groups of the same sex.

Four groups of 9-month-old mice were used for each sex (Table 1). The “No Stressor” group underwent free exploration in the alley and pan, while the “Stressor,” “Stressor and Young Support,” and “Stressor and Old Support” group received the stressor regimen individually, with an 8-week-old, and 9-month-old group, respectively. Thirty minutes after the conclusion of the stressor regimen or free exploration, mice were killed and their trunk blood samples were used for serum CORT assay (Figure 1). A two-way ANOVA revealed that there were significant sex [*F*_(1, 40)_ = 15.51, *p* = 0.0003], treatment [*F*_(3, 40)_ = 62.5, *p* < 0.0001], and sex-treatment interactive [*F*_(3, 40)_ = 2.984, *p* = 0.0425] effects on serum CORT levels in the 9-month-old mice. *Post-hoc* analyses further showed that the stressor regimen significantly enhanced serum CORT levels in male and female mice, while the presence of either young or old same-sex support did not affect such stress-enhanced CORT levels (Figure 6).

## Discussion

In previous studies (Cherng et al., 2010, 2012; Tzeng et al., 2014), we have demonstrated that the present stressor regimen induces decreases in early neurogenesis in the DG, while group housing prevents such stress-induced decreases in 8-week-old male and female Balb/C mice. In this study, however, we found that the same stressor regimen and group housing with the same-sex, age-matched companions did not affect the number of newly proliferated cells or neuroblasts in 8-week-old male or female C57BL/6N mouse DG. These paradoxically conflicting results may arise from mouse strain differences. In fact, it has been documented that adult C57BL/6 mice do not have sex differences in stem cell proliferation or neurogenesis in the DG in a previous report (Lagace et al., 2007). Moreover, Bain et al. (2004) has reported that male C57BL/6 mice do not show any decrease in DG cell proliferation immediately following a 3-h restraint stressor. Furthermore, following a 20-day repeated restraint adaptation, an acute restraint stressor does not seem to change DG cell proliferation in 6-week-old male C57BL/6 mice (Torner et al., 2009).

A previous study has revealed that the presence of companions does not seem to affect the number of newly proliferated cells and neuroblasts in DG of male and female Balb/C mice (Tzeng et al., 2014), mouse free exploring alone served as the baseline condition in this study. The stressor regimen rendered significant decreases in the number of newly proliferated cells and neuroblasts in 9-month-old female, while not in age-matched male, mice, suggesting that middle-aged male mice are resistant but middle-aged female mice are sensitive to stress on early neurogenesis in the DG. The stress resilience findings observed in middle-aged male mice are less likely due to middle-aged male mice' very low baselines in the number of newly proliferated cells and neuroblasts in the DG because group housing significantly decreased the number of newly proliferated cells and neuroblasts in these male mice. Likewise, the sex difference in stress susceptibility should not be attributed to the sex difference in the present stressor regimen-stimulated CORT secretion because both male and female mice exhibited comparable stress-stimulated CORT secretion. Such sex differences in the baseline or stress susceptibility of the dentate neurogenesis have been documented in the literature. For example, we have demonstrated that the present stressor regimen may produce significant decreases in neuronal lineage commitment especially in pro-estrous female mice, but not in male mice (Tzeng et al., 2014). That is, the sex differences in the number of stress-decreased neuroblasts in DG of 9-month-old mice may arise from the possibility that pro-estrous estradiol surge potentiates the stress-stimulated corticosterone secretion and thus results in significant decreases in the number of neuroblasts in females at proestrus (Figueiredo et al., 2007). Although male androgens play a negligible role in modulating the psychosocial stress-induced suppression of cell proliferation (Kambo and Galea, 2006; Buwalda et al., 2010), female estrogens may enhance the number of newly proliferated cells in the DG and stressor-stimulated corticosterone secretion (Tanapat et al., 1999; Ormerod and Galea, 2001; Ormerod et al., 2003; Figueiredo et al., 2007). In fact, female neuroblasts are more prone to self-synthesized GABA to further enhance neurogenesis as compared to male neuroblasts (Yuan et al., 2010). Since neurotransmitters, neuromodulators, microRNAs, and cytokines all have been implicated in the modulation of dentate neurogenesis (Aimone et al., 2014; Woodbury et al., 2015), further study needs to be done to understand the critical molecular substrates and the underlying mechanisms for causing such sex differences in naïve and stress condition.

Regardless of age, we found that group housing prevented the stressor-decreased new cell proliferation and neurogenesis in middle-aged female DG, while such group housing paradoxically potentiated the stress effect by decreasing the number of newly proliferated cells and neuroblasts in middle-aged male DG. Since the presence of young or middle-aged companions did not affect the stress-stimulated CORT secretion in 9-month-old, male or female mice, serum CORT levels might play a minor role in determining such sex differences in the group housing effects. Physical contact has been suspected to play a role in bi-directionally modulating social and drug rewarding effects (Peartree et al., 2012; Tzeng et al., 2016). Unavoidable foot shocks have been reported to induce aggressive behavior in mice (Nath et al., 1982). Since the middle-aged mice received 30-min unavoidable foot shocks with three young or age-matched companions, the middle-aged male mice are suspected to receive more physical contact and/or aggressive attacks compared to the middle-aged female mice in this regard. The quantity and quality of physical contact and/or experimental mice' offensive, defensive attacks throughout the shock delivery protocol are suspected to play a role in differentiating the group housing effect in this regard in male and female mice. Females may obtain larger buffering effects from their social network against the stressor-deteriorating effects on mental health than males (Olstad et al., 2001). Using group housing to model social interaction, we hereby report that group housing appears to be beneficial in buffering the stress impact on dentate neurogenesis in middle-aged female, but not male, mice.

While neurogenesis in the dentate gyrus has been hypothesized to play a critical role in determining the function of hippocampus (Aimone et al., 2014), we found that neither the stressor regimen nor group housing affected the object location or recognition memory performances in the 9-month-old male or female mice. Three mutually related possibilities are provided to explain these negative findings. First, the baselines and stress-caused changes in the number of newly proliferated cells and neuroblasts in middle-aged mouse DG may be too low to provide clear resolutions in the recognition memory performance among control, stress, and stress with support conditions. In these two tasks, control (No Stressor) groups had their recognition ratios slightly higher than 50%, supporting this possibility. Second, the hippocampus-related memory tasks used may be insensitive to the changes in early neurogenesis and the related biochemical environment in the dentate gyrus. It has been estimated that complete neurogenesis and synaptogenesis may take a few weeks in order to make newly formed neurons to display their physiological functions (Aimone et al., 2014). In fact, a recent report documents that perirhinal and medial prefrontal cortices may be more responsible for mediating the object recognition memory (Barker and Warburton, 2015), suggesting that object recognition memory may not be a sensitive behavioral indicator for hippocampal function.

To sum up, we conclude that middle-aged female mice may be more sensitive to the stress-decreasing effects on early neurogenesis in the dentate gyrus as compared with middle-aged male mice. Middle-aged male and female mice are both sensitive to the group housing-exerting stress-modulating effects in this regard. Such sex differences in dentate early neurogenesis in middle-aged mice do not seem to be correlated with their performances in the hippocampus-related memory tasks.

## Author contributions

WT, HW, CGC, and LY were involved in manuscript writing and editing, experimental designs, data collection, and data analysis. CW and JC were responsible for a part of manuscript writing, a part of data collection and data analysis.

## Funding

This study was supported by ROC Ministry of Science and Technology (MOST). ROC MOST had no further role in study design, data collection, data analysis, data interpretation, manuscript writing, or decision to submit this manuscript for publication.

### Conflict of interest statement

The authors declare that the research was conducted in the absence of any commercial or financial relationships that could be construed as a potential conflict of interest.
